# Novel Genes Involved in Hypertrophic Cardiomyopathy: Data of Transcriptome and Methylome Profiling

**DOI:** 10.3390/ijms232315280

**Published:** 2022-12-03

**Authors:** Ivan Kiselev, Maxim Kozin, Natalia Baulina, Maria Pisklova, Ludmila Danilova, Alexandr Zotov, Olga Chumakova, Dmitry Zateyshchikov, Olga Favorova

**Affiliations:** 1 E.I. Chazov National Medical Research Center for Cardiology, Moscow 121552, Russia; 2 Laboratory of Medical Genomics, Pirogov Russian National Research Medical University, Moscow 117997, Russia; 3 Johns Hopkins School of Medicine, Baltimore, MD 21205, USA; 4 Central State Medical Academy of Department of Presidential Affairs, Moscow 121359, Russia

**Keywords:** hypertrophic cardiomyopathy, gene expression, transcriptome, DNA methylation, methylome, pathway analysis

## Abstract

Hypertrophic cardiomyopathy (HCM) is the most common inherited heart disease; its pathogenesis is still being intensively studied to explain the reasons for the significant genetic and phenotypic heterogeneity of the disease. To search for new genes involved in HCM development, we analyzed gene expression profiles coupled with DNA methylation profiles in the hypertrophied myocardia of HCM patients. The transcriptome analysis identified significant differences in the levels of 193 genes, most of which were underexpressed in HCM. The methylome analysis revealed 1755 nominally significant differentially methylated positions (DMPs), mostly hypomethylated in HCM. Based on gene ontology enrichment analysis, the majority of biological processes, overrepresented by both differentially expressed genes (DEGs) and DMP-containing genes, are involved in the regulation of locomotion and muscle structure development. The intersection of 193 DEGs and 978 DMP-containing genes pinpointed eight common genes, the expressions of which correlated with the methylation levels of the neighboring DMPs. Half of these genes (*AUTS2*, *BRSK2*, *PRRT1*, and *SLC17A7*), regulated by the mechanism of DNA methylation, were underexpressed in HCM and were involved in neurogenesis and synapse functioning. Our data, suggesting the involvement of innervation-associated genes in HCM, provide additional insights into disease pathogenesis and expand the field of further research.

## 1. Introduction

Hypertrophic cardiomyopathy (HCM) is the most common inherited heart disease, with an estimated prevalence of about 1:200–1:500, and is characterized by an increase in the wall thickness of the left ventricle (LV) in the absence of obvious loading or metabolic causes for the observed magnitude of myocardial hypertrophy [1,2]. The clinical spectrum of HCM is very broad, from asymptomatic status during long life to progressive heart failure and sudden cardiac death at a young age [1].

HCM is believed to be a classic Mendelian monogenic disease, mostly caused by mutations in sarcomere genes. However, it still remains unrevealed as to what determines the high clinical heterogeneity of the disease, even among carriers of the same mutation and members of the same family, including monozygotic twins [3,4]. Moreover, the rate of identification of pathogenic/likely pathogenic (P/LP) variants in sarcomere or sarcomere-associated genes in HCM patients usually does not exceeds 40–60% [5], and it is still unclear as to what drives the development of HCM in other cases. Furthermore, many loci associated with HCM are also involved in the formation of dilated cardiomyopathy, which is characterized by opposite changes in the LV tissue [6]. These facts gave origin to a new opinion on HCM as an oligogenic disease with a complex inheritance, the phenotype of which is determined via intergenic interactions and the involvement of gene expression regulatory mechanisms at various levels, combined with environmental factors [7]. Recent genome-wide association studies (GWASs) support this hypothesis via the identification of non-sarcomeric HCM-associated loci, which are involved in the modulation of the disease phenotype [8,9]. Additional insights into HCM etiology and its pathogenesis may be provided through an investigation of transcriptome and epigenome changes in the hypertrophied myocardia of HCM patients.

To date, several transcriptomic studies of hypertrophied myocardium have been performed, most of them being in animal models; however, the correlation of the results in the context of HCM pathogenesis is complicated by described differences in gene expression between mouse and human HCM [10]. In humans, genome-wide expression profiles of hypertrophied myocardium were also evaluated [11,12,13,14,15,16]. An investigation into epigenetic modifications that cause long-lasting alterations of gene expression in myocardium can expand the understanding of HCM molecular mechanisms provided by transcriptomic studies, and thus help in elucidating additional genes that contribute to disease development. Such an attempt was performed only in [14], where histone acetylation was analyzed in parallel with the transcriptome. The DNA methylation of CpG dinucleotides in the C5 position of a cytosine ring is one of the most stable and well-studied epigenetic modifications that regulate the expression of the nearest genes through different mechanisms [17]. There are two studies of DNA methylation in the LV tissues of HCM patients that have been performed to date [18,19].

In the present study, we aimed to identify novel HCM-driving genes by employing a dual-omics approach, which includes RNA sequencing and genome-wide DNA-methylation analysis. As a comparison group, patients with aortic stenosis (AS) were used, in whom secondary LV hypertrophy develops compensatorily to maintain wall stress and cardiac function in the conditions of the increased afterload. By this, we managed to measurably level out the contribution of biological processes participating in the hypertrophy pathogenesis, regardless of its origin (such as cardiac energy balance, response to oxidative stress, apoptosis, fibrosis, etc.), and thus, to point towards new genes that may play an important role in regulating the pathological mechanisms underlying HCM.

## 2. Results

### 2.1. Clinical Characteristics of the Studied Individuals

A total of 13 HCM and 14 AS patients undergoing surgical interventions were enrolled in the study. The discovery sample consisted of eight HCM and five AS patients, while the overall sample was used for the RT-qPCR validation of the findings.

The main clinical characteristics of all of the studied patients are shown in Table 1 and Table S1. The maximal LV wall thickness differed significantly between the studied groups (Mann–Whitney *p*-value < 0.05) in the overall and discovery samples. In the overall sample, differences in left atrium diameter, end-systolic volume index, Sokolow-Lyon index, and level of NT-proBNP between HCM and AS were significant.

### 2.2. Analysis of Gene Differential Expression Using RNA Sequencing

RNA sequencing (RNA-seq) was performed in LV myocardial samples of eight HCM and five AS patients (Table 1 and Table S1). Principal component analysis after variance stabilizing transformations showed that not only disease status, but also sex (at least in case of HCM) was among the main sources of variability between the samples (Figure S1). Therefore, sex was included in the design formula in DESeq2 analysis as a covariate. Levels of 20887 transcripts were analyzed.

**Figure 1:**
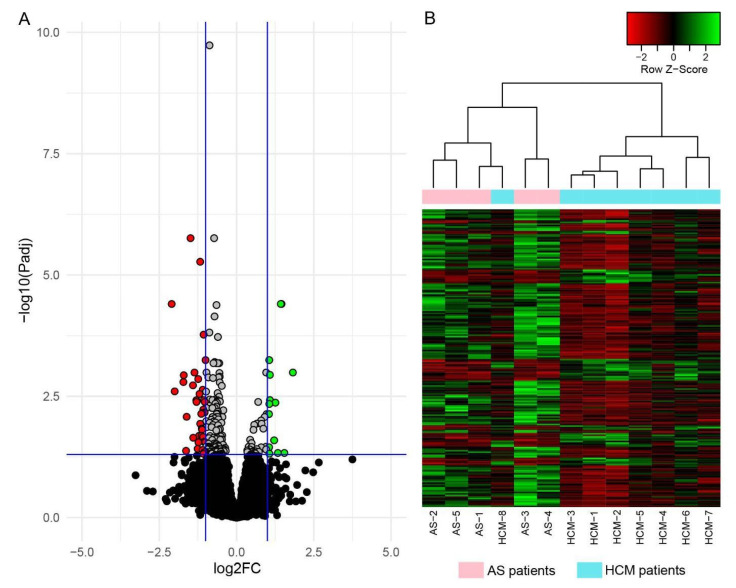
Visualization of the DEGs identified in the LV myocardial tissue of HCM patients when compared to AS patients. (**A**): Volcano plot of gene expression levels. Blue vertical lines indicate threshold for expression differences |log2FC| = 1; blue horizontal line—threshold for statistical significance (padj = 0.05). Significantly downregulated genes (padj < 0.05, log2FC < −1) are marked in red; significantly upregulated genes (padj < 0.05, log2FC > 1)—in green; genes with padj < 0.05 that do not exceed the threshold |log2FC| = 1—in gray. (**B**): Heatmap of z-scored expression levels of 193 identified DEGs in all studied patients. Decreased expression levels of DEGs are marked in red, and increased levels—in green. Top dendrogram shows hierarchical clustering of RNA samples. Cyan indicates RNA samples from HCM patients; pink—from AS patients.

We found 193 genes, the expression levels of which significantly differed (padj < 0.05) between HCM and AS patients (Figure 1A and Table S2). A total of 149 (77.2%) of these differentially expressed genes (DEGs) were shown to be downregulated in HCM. A heatmap of DEG expression levels in all participants of the study is shown in Figure 1B. A hierarchical clustering of RNA samples showed that HCM and AS patients aggregated into separate clusters, with the only exception being the patient HCM-8, who had the smallest LV wall thickness among all of the HCM patients.

When considering DEGs via the most pronounced changes in expression between HCM and AS patients (|Log2FC| > 1), 38 genes were characterized by lower levels in HCM patients (Log2FC from −1.00 to −2.10), and 14 genes—by higher levels (Log2FC from 1.05 to 1.82) (Table 2).

### 2.3. Genome-Wide DNA Methylation Analysis

To search for the DNA methylation patterns specific for the myocardium of HCM patients, we performed genome-wide DNA methylation profiling of the same LV samples obtained from eight HCM and five AS patients (see Table 1 and Table S1). Methylation data from 729,969 individual CpG sites passing filtering were included in the analysis. SVD analysis showed that subjects’ sex and age, as well as the batch effects of microarrays, do not significantly contribute to the observed variance (Figure S2); thus, we did not include them in the further analysis as covariates.

**Figure 2:**
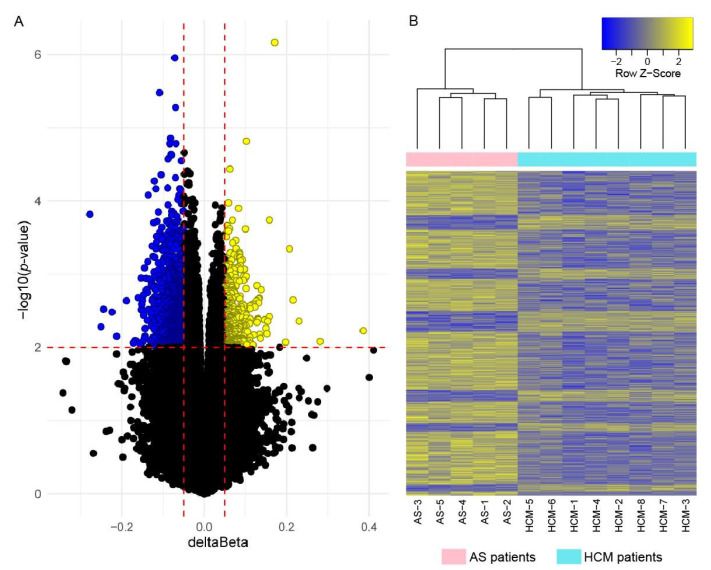
Visualization of the DMPs identified in LV myocardial tissue of HCM patients when compared to AS patients. (**A**): Volcano plot of all CpG site methylation levels. Red vertical lines indicate threshold for methylation differences (|deltaBeta| = 0.05); red horizontal line—threshold for statistical significance (*p* = 0.01). DMPs passing both thresholds are marked on the figure with blue dots (hypomethylated in HCM) and yellow dots (hypermethylated in HCM). (**B**): Heatmap of z-scored methylation levels of 1755 identified DMPs in all studied patients. Methylation level varies from fully unmethylated (blue) to fully methylated (yellow). Top dendrogram shows hierarchical clustering of DNA samples. Cyan indicates DNA samples from HCM patients; pink—from AS patients.

No differentially methylated positions (DMPs) crossed the genome-wide significance threshold *p* ≤ 5 × 10^−8^. Using a nominal significance threshold (*p* < 0.01, deltaBeta > 0.05), we detected 1755 DMPs (Figure 2A and Table S3). A heatmap of DMP methylation levels is shown in Figure 2B. The hierarchical clustering of DNA samples on a heatmap demonstrated their well-defined aggregation into separate clusters corresponding to HCM and AS patients. In total, 1367 DMPs (77.9% of total) were hypomethylated in HCM; 1107 DMPs (63.1% of total) were located in/near 978 known genes.

### 2.4. Correlation of Gene Expression and DNA Methylation Data

**Figure 3:**
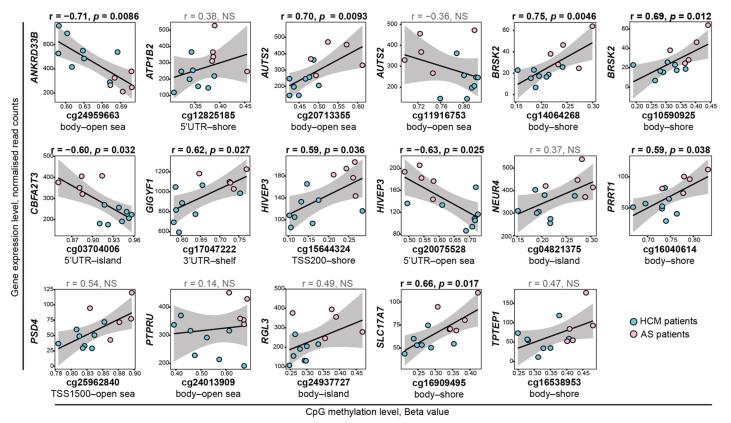
Correlation of expression of 14 DEGs with methylation levels of 17 neighboring DMPs. Cyan and pink dots represent HCM and AS patients, respectively. The black regression line and its confidential interval (gray area) are shown on each plot. Spearman’s rank correlation coefficients and *p*-values calculated for each of the 17 DEG–DMP pairs are presented above corresponding dot plots. Significant coefficients and *p*-values are marked in bold. Location of DMPs according to gene functional elements and CpG landmarks are indicated under each plot. TSS1500 and TSS200—regions within 1500-200 and 200-0 nucleotides upstream from the transcription start site (TSS), respectively; 5’UTR and 3’UTR—5′ and 3′ untranslated regions, respectively. NS—not significant.

In order to search for the possible effects of DNA methylation levels on the expression of neighboring genes, we matched the results obtained by RNA-seq and methylation analyses. Intersecting the lists of 193 DEGs and 978 DMP-containing genes identified 14 genes that were both differentially expressed in HCM and contained DMPs. Spearman’s correlation analysis (Figure 3) identified significant correlations of gene expression and DNA methylation levels for 8 out of 14 genes, namely *ANKRD33B*, *AUTS2*, *BRSK2*, *CBFA2T3*, *GIGYF1*, *HIVEP3*, *PRRT1*, and *SLC17A7*. The expression levels of *BRSK2* and *HIVEP3* correlated with the methylation levels of two DMPs. The expression of *HIVEP3* correlated positively with methylation level of cg15644324 (r = 0.59, *p* = 0.036) and negatively—with methylation level of cg20075528 (r = −0.63, *p* = 0.025). The expression of *BRSK2* positively correlated with methylation levels of both cg14064268 and cg10590925 (r = 0.74 and 0.68, *p* = 0.0046 and 0.018, respectively). Other positive correlations were observed between the expression of *AUTS2*, *GIGYF1*, *PRRT1*, and *SLC17A7;* and the methylation levels of cg20713355, cg17047222, cg16040614, and cg16909495, respectively (r from 0.70 to 0.59, p from 0.0093 to 0.038). Negative correlations were identified between *ANKRD33B* and *CBFA2T3,* and cg24959663 and cg03704006, respectively (r = −0.71 and −0.60, and *p* = 0.0086 and 0.032, respectively).

### 2.5. RT-qPCR Validation

**Figure 4:**
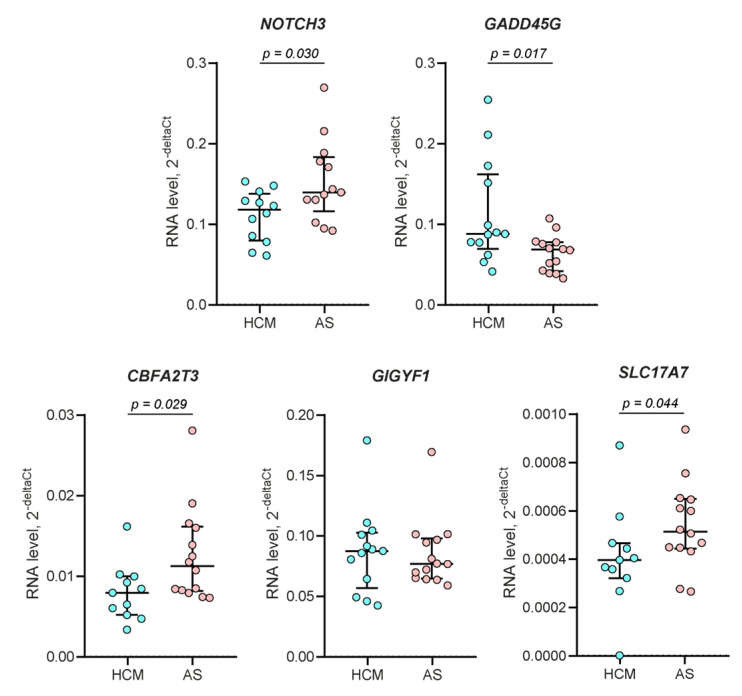
Expression levels of *NOTCH3*, *GADD45G*, *GIGYF1*, *SLC17A7*, and *CBFA2T3* in 13 HCM patients and 14 AS patients, as determined via RT-qPCR. RNA levels were calculated relative to *GAPDH*. The data are presented using a scatter plot (median with interquartile ranges). The differences in RNA levels between HCM patients and AS patients were considered to be significant if the *p*-value in the Mann–Whitney U test was less than 0.05.

In order to confirm high-throughput sequencing data via independent methods, we evaluated the expression levels of several genes using RT-qPCR in the overall samples of 13 HCM patients and 14 AS patients (see Table 1 and Table S1). *NOTCH3* and *GADD45G* were randomly selected among the top five genes that were down- and upregulated in HCM, respectively, and *CBFA2T3*, *GIGYF1*, and *SLC17A7* were selected among genes with correlated expression and DNA methylation levels. Expression changes were confirmed for all genes, with the only exception being for GIGYF1, the level of which did not differ significantly between the studied groups (Figure 4).

### 2.6. Gene Ontology Enrichment Analysis

In order to evaluate functional patterns affected by 193 DEGs and 978 DMP-containing genes, we performed gene ontology (GO) enrichment analysis in the “biological process” category. DEGs were significantly enriched in 83 GO terms (Table S4), and DMP-containing genes in 285 GO terms (Table S5). Sixty-four biological processes were overrepresented by genes from both sets, accounting for 77.1% of all DEG-enriched GO terms and 22.5% of GO terms enriched by DMP-containing genes (Table S6).

**Figure 5:**
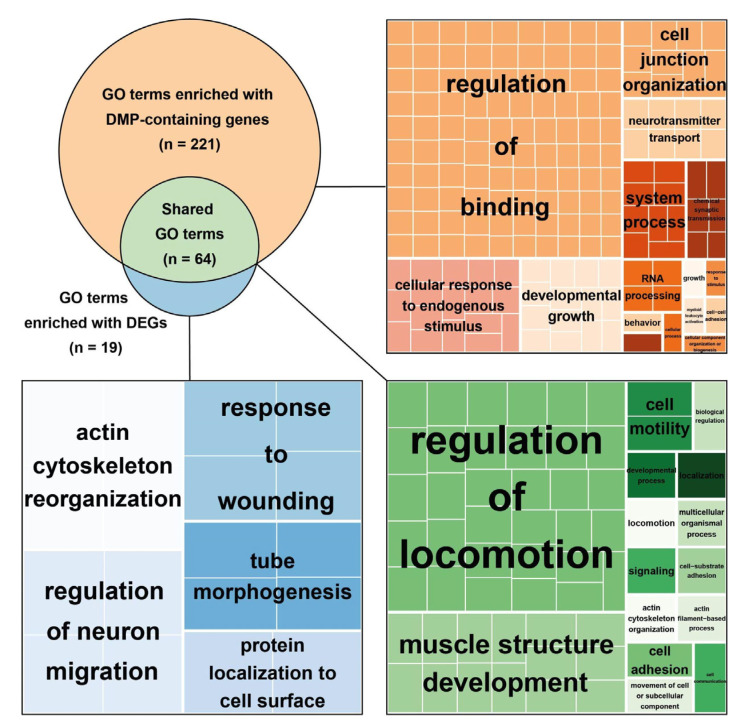
Visualization of GO terms overrepresented by DEGs and DMP-containing genes. Top left Euler-Venn diagram schematically represents sets of identified GO terms enriched with DMP-containing genes (orange) or with DEGs (blue) and their overlap (green). TreeMap diagrams show REVIGO clasterization of the identified GO term sets by similarity. Each colored rectangle on the map represents one GO term; the size of the rectangle indicates the ‘uniqueness’ of the GO term—the negative of its average similarity to all other input terms. Groups of similar processes forming the cluster are marked with the same color.

The clustering of identified biological processes using REVIGO software (Figure 5) showed that the majority of GO terms, overrepresented by both DEGs and DMP-containing genes, were associated with the regulation of locomotion and muscle structure development. Biological processes enriched only with DEGs are involved in the regulation of neuron migration, and in cytoskeleton organization and functioning, while processes enriched only by DMP-containing genes are involved in the regulation of binding and cell junction organization, and in responses to endogenous stimuli and neurotransmitter transport.

## 3. Discussion

Although HCM was described several decades ago, the understanding of its etiology and pathogenesis still evolves. New data obtained using high-throughput genomic methods refine the picture on the molecular mechanisms underlying this common disease with significant social consequences. The results of the present study expand a limited series of transcriptome and DNA methylome investigations performed in the heart tissues of HCM patients.

In order to advance the understanding of what processes are involved in the formation of primary LV hypertrophy, we compared the transcription profiles of patients with HCM and patients with AS. Although AS patients also developed LV hypertrophy as a result of a long-lasting overloading condition, it was much less pronounced in AS than in HCM patients (the maximal LV wall thickness was 15.0 ± 2.0 and 23.5 ± 5.6 mm, respectively; *p* = 0.0039). We identified significant differences in myocardium transcription profiles between HCM and AS patients: 193 DEGs passed the threshold for multiple corrections, and most of them were underexpressed in HCM. When considering only 52 DEGs with the most pronounced changes in expression between HCM and AS (|Log2FC| > 1), C4B was shown to be the most significantly downregulated gene in HCM; it encodes complement 4b, which is abundantly expressed in the healthy heart muscle [20] and was previously shown to be downregulated in the peripheral blood plasma of HCM patients [21]. *NOTCH3*, a top-two downregulated gene in HCM, also attracts attention since it is well known to play an essential role in the protection of heart tissue from apoptosis and fibrotic changes [22,23,24]. Too little is currently known about the most upregulated genes, *EIF4EBP3* and *CTXND1*; their role in cardiac tissue still remains to be uncovered. The expression level of the top-three upregulated gene, *GADD45G*, negatively correlates with LV reverse remodeling [25]. In general, the most significant DEGs participate in the processes of the inflammation and regeneration of the heart; the levels of *NOTCH3* and *GADD45G* in the HCM group, in comparison with AS, may indicate decreased compensatory abilities of the primary hypertrophied myocardium.

An important benefit of our study was in the fact that the DNA methylation profiling was performed in addition to transcriptome profiling in the same myocardium samples from HCM and AS patients. We showed that the profile of DMPs clearly distinguished HCM patients from AS patients, most likely indicating the involvement of DNA methylation in the formation of primary LV hypertrophy.

By comparing the lists of DEGs and genes containing DMPs, we identified a significant correlation of the expression of eight genes with methylation levels of the neighboring DMPs; these genes can be involved in the formation of the HCM clinical phenotype. Half of these genes—*AUTS2*, *BRSK2*, *PRRT1*, and *SLC17A7*—are implicated in neurodevelopment and synapse formation, suggesting differences in heart innervation between HCM and AS patients. *AUTS2* is highly expressed in the brain and mostly plays a role in transcription regulation in neurons during development [26]. *BRSK2* encodes serine/threonine kinase *SAD-A*, which intermediates external signals, polarizing neurons and promoting axonogenesis [27]. The *PRRT1* gene product slows the deactivation and desensitization of the synaptic AMPA-regulated glutamate receptors, which are necessary for synapse development and function [28]. *SLC17A7* (also known as *VGLUT1*) encodes a multifunctional transporter mainly studied in the neuron-rich regions of the brain, where it transports L-glutamate from the cytoplasm into synaptic vesicles at presynaptic nerve terminals [29]. The decreased expression of the above-mentioned genes in HCM was positively correlated with the hypomethylation of CpG sites in gene bodies. This phenomenon is not surprising, since gene body methylation is known to be mostly associated with gene expression upregulation [30]. The remaining identified genes play roles in tyrosine kinase receptor signaling (*GIGYF1*) and transcription regulation (*CBFA2T3* and *HIVEP3*), or they have unknown functions (*ANKRD33B*).

Gene Ontology enrichment analysis identified a broad variety of biological processes significantly enriched by DEGs (83 pathways) and by DMP-containing genes (285 pathways). The majority of GO terms enriched by genes from both gene sets are associated with the regulation of locomotion and muscle structure development (see Figure 4), which points toward the differences in the molecular mechanisms of hypertrophy formation in HCM and AS, both at the transcriptomic and epigenomic levels.

Interestingly, a REVIGO analysis highlighted quite a large cluster of GO terms such as “regulation of neuron migration” enriched by DEGs and a cluster “neurotransmitter transport” enriched by DMP-containing genes. Although similar innervation-associated clusters were not observed among shared GO terms, pathway GO:0022008 “neurogenesis” was identified during a manual search (see Table S6). Thus, GO terms associated with neuron functioning were enriched by both DEGs- and DMP-containing genes. This fact supports the assumption on the differences in heart innervation between HCM and AS patients, which was previously made based on the functions of genes identified in a correlation analysis. As expected, two of these genes, *AUTS2* and *BRSK2*, participate in the pathway GO:0022008 “neurogenesis”, mentioned above. Several other genes associated with cardiomyopathies were earlier shown to be involved in a variety of neuromuscular disorders [31]; this points additionally to the link between innervation-related mechanisms and heart pathology. Sympathetic and parasympathetic innervation of the heart is known to play a crucial role in heart maturation during ontogenesis, through regulation of the proliferation, physiological hypertrophy, metabolism, and several electrophysiologic properties of cardiomyocytes [32]. In adult hearts, sympathetic/parasympathetic imbalance is associated with pathological remodeling and heart failure [33,34]. An innervation imaging may have a prognostic value in patients with cardiomyopathy [35]. Overall, these data suggest the involvement of innervation-associated mechanisms in HCM pathogenesis.

In contrast to previous transcriptomic studies searching for the differences between HCM myocardium and healthy heart tissue, we used samples from AS patients as a control. However, it seems that the pattern of biological processes affected by identified DEGs is quite similar to previous studies. Indeed, the main biological pathways previously shown to be deregulated in HCM were associated with cell motility, muscle cell development, cytoskeleton reorganization and adhesion, angiogenesis, response to wounding, etc. [11,13,14]; similar GO terms were highlighted in the present work (see Table S4). The processes involved in neurogenesis and neuromuscular disorders were also reported [11,14], however, they did not receive much attention. At the same time, in contrast to published studies [14,16], we did not observe the dysregulation of pathways affecting ATP synthesis and mitochondrial functions in HCM. Most likely, these processes are involved not only in the pathogenesis of HCM, but also in AS-associated secondary LV hypertrophy. Thus, by using AS the comparison group, we were unable to identify any and all aspects of HCM pathogenesis, but we revealed the processes differently involved in genetically driven myocardial hypertrophy in HCM, and the more common secondary hypertrophy instead.

Our study included HCM patients with identified P/LP variants in the *ALPK3* gene (the patient HCM-2) and the *MYBPC3* gene (the patient HCM-5), along with six genotype-negative patients. However, the DEG profiles of HCM patients (except for the patient HCM-8), as well as their DMP profiles (without exception) clustered together (see Figure 1 and Figure 2). The published data also show that gene expression changes are mostly similar among HCM patients with P/LP variants in different causative genes [12]. Altogether, it suggests the existence of the universal molecular mechanisms of HCM pathogenesis, regardless of the differences in genetic background.

By choosing AS patients as a comparison group for our study, we were able to exclude the confounding effects of universal LV hypertrophy pathogenic factors, such as arterial hypertension, which was equally prevalent in HCM and AS patients (84.6% and 85.7% of patients, respectively). However, adding the data on transcriptome and methylome profiling in healthy heart samples would certainly expand the informativity of the research. In this connection, we have to say that the inaccessibility of healthy LV tissues for subsequent analysis is the discernible limitation of our study. Another limitation is the small number of participants, which is determined by the number of surgical interventions performed; it entails a lack of statistical power to discover genome-wide DNA methylation changes. Nevertheless, the prominent similarity of the biological processes affected by DEGs and DMPs in our study and the processes reported in previous works, in our opinion, testify to the validity of our data.

Generally, we observed clear differences in the gene expression and DNA methylation profiles of LV myocardial samples obtained from HCM and AS patients, which indicate the differences in the molecular mechanisms that define primary and secondary myocardial hypertrophy. Such a multilevel analysis pinpointed eight genes involved in HCM, which might be the subjects of further study. We believe that our data, suggesting the possible involvement of innervation-associated genes in HCM, can provide additional insights into disease pathogenesis and expand the field of research.

## 4. Materials and Methods

### 4.1. Patients and Controls

Thirteen patients with severe obstructive HCM (mean age 56.5 ± 12.0 years; women 46.2%) were recruited into the study. HCM was diagnosed based on the 2014 recommendations of the European Society of Cardiology [36]: increased LV wall thickness ≥ 15 mm that was not solely explained by abnormal loading conditions. Patients suspicious of phenocopies of HCM were excluded from the study. Other exclusion criteria were systemic autoimmune, viral, bacterial, and oncological diseases. All HCM patients were previously studied by a targeted massively parallel sequencing method using a panel that included robust validated HCM-associated genes and the genes of its main phenocopies (*ACTC1*, *DES*, *FHL1*, *FHOD3*, *GLA*, *LAMP2*, *MYBPC3*, *MYH7*, *MYL2*, *MYL3*, *PRKAG2*, *PTPN11*, *TNNC1*, *TNNI3*, *TNNT2*, *TPM1*, *TRIM63*, and *TTR*) in certified genetic laboratories. One HCM patient carried a variant in the *MYBPC3* gene, and one patient in the *ALPK3* gene; in other patients, P/LP variants were not identified. The control group consisted of 14 patients with severe AS (mean age 60.4 ± 9.0 years; women 35.7%).

### 4.2. Sample Processing

Myocardial samples were obtained from HCM and AS patients undergoing surgical interventions. In HCM patients, the sampling area was located under the right aortic coronary leaflet and corresponded to the zone of Morrow septal myectomy. In AS patients, myocardial biopsies sized 5 × 7 × 10 mm were taken from the left-side interventricular septum during open-heart surgery before aortic valve replacement. All interventions were performed in the Federal Scientific and Clinical Center of Specialized Medical Care and Medical Technologies (Moscow, Russia) by the same surgeon. After extraction, tissue samples were immediately cut into fragments up to ≤ 0.5 cm in any single dimension, submerged in 5 volumes of RNAlater solution (Thermo Fisher Scientific, Waltham, MA, USA), and stored at -20 °C for further processing. The disruption of tissue samples was performed using the TissueLyser LT bead mill (Qiagen, Germantown, MD, USA). Total RNA was extracted from disrupted samples using the RNeasy Mini Kit (Qiagen, USA), and genomic DNA using the DNA Mini Kit (Qiagen, USA). RNA and DNA purity and quantity were assessed using the NanoDrop 2000 spectrophotometer (Thermo Fisher Scientific, USA). RNA integrity was assessed by the QIAxcel Advanced System (Qiagen, USA); samples with RNA integrity numbers of above eight were included in subsequent experiments.

### 4.3. RNA-Seq Analysis

RNA libraries were constructed from 1 μg RNA using MGIEasy RNA Library Prep Set following manufacturer instructions. Global transcriptome profiling was performed via high throughput RNA-seq on the MGISEQ-200 platform.

The quality control of raw sequencing reads was assessed with FASTQC. Low quality nucleotides were trimmed from the raw fastq files using Trimmomatic v. 0.39. Samples were mapped on Genome primary assembly GRCh38 with the Gencode annotation using STAR v. 2.7.6a [37]. Reads per gene were counted using the GeneCounts option of the STAR software. Genes with a small number of reads (<100 per sample) were excluded from the analysis.

DESeq2 R package v. 1.32.0 was used for normalization, principal component analysis, and differential gene expression analysis (design = ∼ Sex + Condition) [38]. The amplitude of expression changes was represented in the log2FC format. The False Discovery Rate (FDR) procedure of Benjamini-Hochberg was used to adjust for multiple testing corrections [39].

### 4.4. Genome-Wide DNA Methylation Analysis

Bisulfite conversion of the genomic DNA was performed using the EZ DNA Methylation-Gold Kit (Zymo Research, Irvine, CA, USA). DNA methylation levels were analyzed on the Infinium MethylationEPIC BeadChip using the iScan array scanner (Illumina, USA) at the SB RAS Genomics Core Facility (Institute of Chemical Biology and Fundamental Medicine SB RAS, Novosibirsk).

Methylation data processing and normalization were performed using the ChAMP R package v. 3.14 [40]. Probes with detection *p*-values above 0.01, multi-hit probes, probes close to SNPs, and non-autosomal probes were filtered out from the subsequent analysis using the champ.filter() function with default parameters. A beta-mixture quantile normalization method (BMIQ) [41] provided by the champ.norm() function was used to normalize methylation data for probe type bias (quality control plots for non-normalized and normalized methylation data are provided in the Figure S3). The examination of the methylation data for possible covariates, such as sex and age, as well as the batch effects of different microarray slides, was performed using singular value decomposition (SVD) analysis implemented in the champ.SVD() function. The evaluation of methylation level for each probe was performed by calculating its beta-value—the ratio of the methylation signal intensity and the overall intensity (the sum of intensities of the methylated and unmethylated probes). Beta-values ranged for each probe from 0 (fully unmethylated probes) to 1 (fully methylated probes). DMPs were identified using the general linear model. Along with the standard *p*-value, FDR correction was applied.

### 4.5. RT-qPCR

Total RNA was reverse transcribed using the High Capacity RNA-to-cDNA Kit (ThermoFisher Scientific, USA), and cDNA was then subjected to qPCR in triplicate using the TaqMan Gene Expression Assays (ThermoFisher Scientific, USA) according to the manufacturer’s protocol. *GAPDH* was used as an endogenous control. The relative expression of each gene in the sample was calculated as 2-deltaCt, where deltaCt for the gene is equal to its average Ct minus the average Ct of *GAPDH*. The comparison of gene expression levels between groups was performed using the Mann–Whitney U test (*p* < 0.05 were considered significant).

### 4.6. Gene Set Enrichment Analysis and Data Visualization

GO term enrichment analysis was performed using the Panther classification system [42]. GO terms with FDR corrected *p*-values of less than 0.05 were considered as being significant. In order to shorten the list of GO terms and to find the most representative ones, we used the REVIGO service, which implements a simple clustering algorithm based on measures of semantic similarity and the following redundancy reduction; a reduction setting of “Large (0.9)” was used for the analysis [43].

Ggplot2 v. 3.3.5, gplots v. 3.1.1, and Treemap 2.4–3 R packages [44,45,46], as well as the standard tools of the R environment were used for data visualization.

## Figures and Tables

**Table 1:** Characteristics of HCM and AS patients involved in the study.

Characteristics	Overall Sample		Discovery Sample	
	HCM, N = 13	AS, N = 14	HCM, N = 8	AS, N = 5
Age, years	56.5 ± 12.0	60.4 ± 9.0	55.0 ± 12.7	64.0 ± 9.5
Female, n (%)	6 (46.2)	5 (35.7)	4 (50.0)	1 (20.0)
BMI, kg/m^2^	28.7 ± 4.3	28.1 ± 4.0	30.6 ± 3.5	29.2 ± 5.4
Atrial fibrillation, n (%)	3 (23.1)	2 (14.3)	2 (25.0)	1 (20.0)
Ventricular tachycardia, n (%)	5 (38.5)	4 (28.6)	2 (25.0)	1 (20.0)
Arterial hypertension, n (%)	11 (84.6)	12 (85.7)	6 (75.0)	5 (100.0)
Coronary heart disease, n (%)	5 (38.5)	4 (28.6)	4 (50.0)	4 (80.0)
Diabetes mellitus, n (%)	2 (15.4)	4 (28.6)	2 (25.0)	1 (20.0)
Data of instrumental examination and laboratory tests				
Maximal LV wall thickness, mm	**22.5 ± 5.0**	**16.6 ± 3.3**	**23.5 ± 5.6**	**15.0 ± 2.0**
LA diameter, mm	**44.5 ± 3.7**	**40.7 ± 4.6**	44.1 ± 3.5	41.4 ± 2.3
LA end-systolic volume index, mL/m^2^	**49.9 ± 11.3**	**40.8 ± 9.6**	49.3 ± 11.1	38.8 ± 9.5
Maximal LV outflow tract pressure gradient, mmHg	114.6 ± 27.6	100.8 ± 28.5	122.1 ± 28.4	90.0 ± 35.8
LV ejection fraction, %	61.4 ± 6.9	55.8 ± 7.4	62.3 ± 8.3	58.0 ± 5.1
Severe mitral regurgitation, n (%)	5 (38.5)	0 (0.0)	4 (50.0)	0 (0.0)
Giant T-wave inversions, n (%)	4 (30.8)	0 (0.0)	2 (20.0)	0 (0.0)
Sokolow-Lyon index, mm	**39.5 ± 13.5**	**29.6 ± 10.6**	38.3 ± 15.4	27.2 ± 10.8
eGFR, mL/min	99.0 ± 40.6	99.0 ± 33.1	108.8 ± 41.0	87.1 ± 37.3
NT-proBNP, pg/ml	**1646.6 2± 1497.2**	**728.6 ± 1462.6**	1901.6 ± 1757.8	348.4 ± 356.5
Drug administration				
Beta-blockers, n (%)	12 (92.3)	9 (64.3)	7 (87.5)	5 (100.0)
ACE inhibitors or ARBs, n (%)	10 (76.9)	8 (57.1)	6 (75.0)	4 (80.0)
Loop diuretics, n (%)	6 (46.2)	7 (50.0)	4 (50.0)	4 (80.0)
MRAs, n (%)	3 (23.1)	3 (21.4)	2 (25.0)	2 (40.0)

Parameters are marked in bold if they significantly differ between HCM and AS patients in the sample (Mann–Whitney *p*-value < 0.05). ACE—angiotensin-converting enzyme, AS—aortic stenosis, ARB—angiotensin receptor blocker, BMI—body mass index, eGFR—estimated glomerular filtration rate, HCM—hypertrophic cardiomyopathy, LA—left atrium, LV—left ventricle, MRA—mineralocorticoid receptor antagonist, NT-proBNP—N-terminal pro–B-type natriuretic peptide.

**Table 2:** Differentially expressed genes identified in the LV myocardial samples of patients with HCM when compared to AS (padj < 0.05, |Log2FC| > 1).

No.	Gene	Genomic Location	Log2 FC	*p*_adj_-Value	No.	Gene	Genomic Location	Log2 FC	*p*_adj_-Value
Genes downregulated in HCM									
1	*C4B*	6p21.33	−1.49	1.74 × 10^−6^	20	*PTGIR*	19q13.32	−1.10	0.0070
2	*NOTCH3*	19p13.12	−1.18	5.33 × 10^−6^	21	*BGN*	Xq28	−1.14	0.0073
3	*IGF2*	11p15.5	−2.10	3.95 × 10^−5^	22	*GRAMD1C*	3q13.31	−1.61	0.0084
4	*LAMA5*	20q13.33	−1.06	0.00017	23	*NACA*	12q13.3	−1.17	0.012
5	*LTBP4*	19q13.2	−1.00	0.00057	24	ENSG00000272789.1	2q14.3	−1.12	0.015
6	*C4A*	6p21.33	−1.36	0.0010	25	*SSPOP*	7q36.1	−1.11	0.015
7	*LMX1B*	9q33.3	−1.71	0.0012	26	*CCDC80*	3q13.2	−1.21	0.021
8	*PKD1P4*	16p12.3	−1.24	0.0014	27	*MN1*	22q12.1	−1.13	0.021
9	*SLC35F2*	11q22.3	−1.72	0.0016	28	*MRC2*	17q23.2	−1.08	0.022
10	*SPOCK1*	5q31.2	−1.41	0.0019	29	*GPC6*	13q31.3	−1.40	0.023
11	*ITGA11*	15q23	−1.09	0.0023	30	*NR1D1*	17q21.1	−1.04	0.027
12	*UCKL1-AS1*	20q13.33	−2.00	0.0025	31	*SLC6A9*	1p34.1	−1.23	0.028
13	*KCNC3*	19q13.33	−1.20	0.0028	32	*PSD4*	2q14.1	−1.01	0.028
14	*NECTIN1*	11q23.3	−1.06	0.0038	33	*ADAMTS5*	21q21.3	−1.26	0.038
15	*BRSK2*	11p15.5	−1.30	0.0038	34	*TPTEP1*	22q11.1	−1.10	0.040
16	*CHGB*	20p12.3	−1.29	0.0041	35	*NDUFA13*	19p13.11	−1.63	0.042
17	*KCNT1*	9q34.3	−1.03	0.0041	36	*MYH11*	16p13.11	−1.00	0.045
18	*CCN3*	8q24.12	−1.08	0.0060	37	*PTK7*	6p21.1	−1.04	0.045
19	*NCOR2*	12q24.31	−1.03	0.0068	38	*DPT*	1q24.2	−1.07	0.049
Genes upregulated in HCM									
1	*EIF4EBP3*	5q31.3	1.45	3.95 × 10^−5^	8	ENSG00000279041.1	8p12	1.06	0.0046
2	*CTXND1*	15q25.1	1.43	3.95 × 10^−5^	9	*APOD*	3q29	1.05	0.0074
3	*GADD45G*	9q22.2	1.06	0.00057	10	ENSG00000287047.1	10q25.1	1.22	0.026
4	*ATRNL1*	10q25.3	1.82	0.0010	11	*SLC26A4*	7q22.3	1.05	0.036
5	*ST8SIA5*	18q21.1	1.08	0.0011	12	ENSG00000286401.1	10q11.23	1.34	0.047
6	*SOCS2-AS1*	12q22	1.08	0.0038	13	*C2CD6*	2q33.1	1.55	0.047
7	*SH3GL2*	9p22.2	1.26	0.0043	14	*APOA1*	11q23.3	1.06	0.048

## Data Availability

RNA-seq data are available in the Gene Expression Omnibus (GEO) database under accession number GSE206978, and genome-wide DNA methylation data—under number GSE207095.

## Supplementary Materials

The following supporting information can be downloaded at: https://www.mdpi.com/article/10.3390/ijms232315280/s1, Figure S1: Two-dimensional clustering of samples based on principal component analysis of 500 most variable genes. Percentage of variance explained by each principal component (PC) is indicated on the corresponding axis; Figure S2: Singular value decomposition analysis (SVD) plots for normalized methylation data from 729969 probes passing filtering; Figure S3: Quality control plots for non-normalized and normalized methylation data from 729969 probes passing filtering; Table S1: Characteristics of HCM and AS patients included in the study; Table S2: Differentially expressed genes identified in myocardia of HCM patients when compared to AS (padj < 0.05); Table S3: Differentially methylated CpG sites identified in myocardia of HCM patients when compared to AS (*p* < 0.01, delta β > 0.05); Table S4: GO Biological processes significantly enriched (adj. *p* < 0.05) by differentially expressed genes (DEGs), which were identified in the myocardia of HCM patients; Table S5: GO Biological processes significantly enriched (adj. *p* < 0.05) by genes containing differentially methylated positions (DMPs), which were identified in the myocardia of HCM patients; Table S6: GO Biological processes significantly enriched (adj. *p* < 0.05) by both differentially expressed genes (DEGs) and genes containing differentially methylated positions (DMPs).
