# Supplementary material for: Novel Genes Involved in Hypertrophic Cardiomyopathy: Data of Transcriptome and Methylome Profiling
Source: Int J Mol Sci. 2022 Dec 3;23(23):15280. doi: 10.3390/ijms232315280 (PMC9739701; doi:10.3390/ijms232315280)
Supplement: Supplementary file 1 [file ijms-23-15280-s001.zip › Figures_S1-S3.pdf]

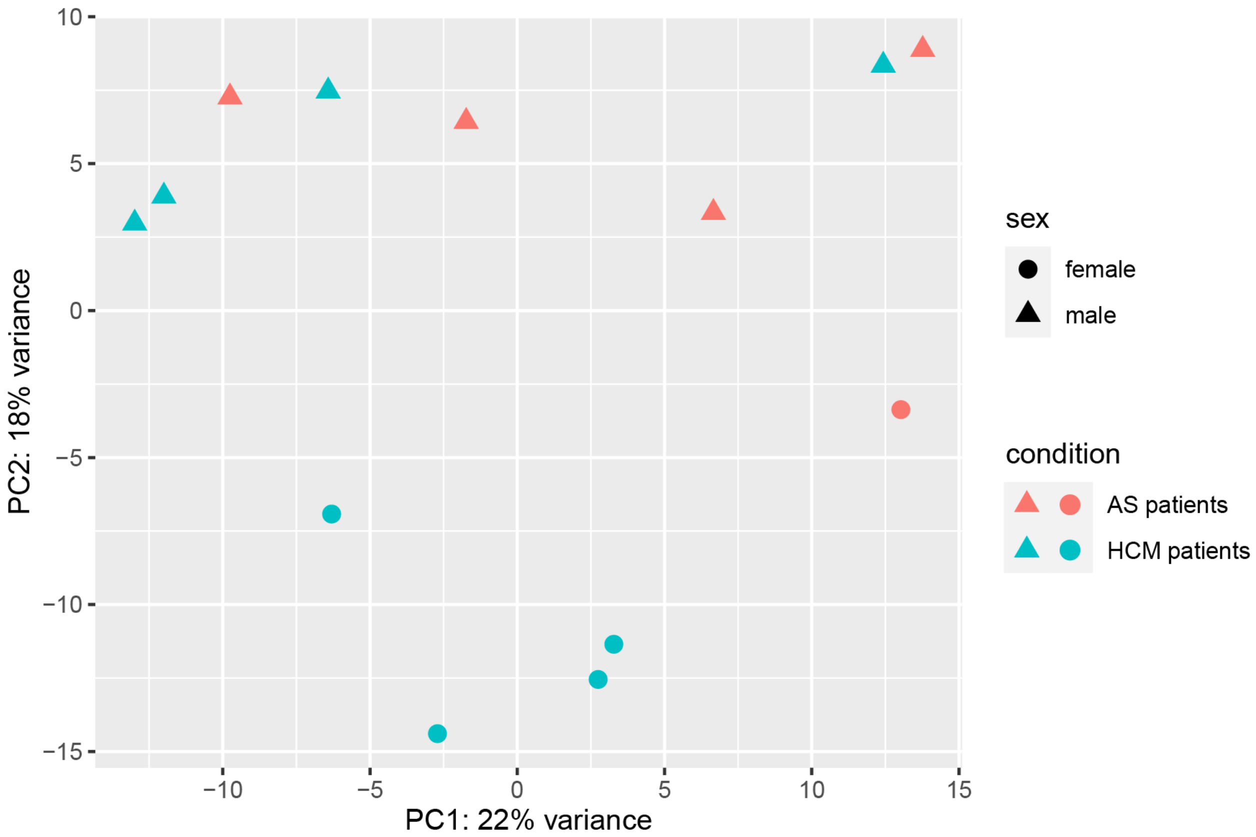

Figure S1. Two-dimensional clustering of samples based on principal component analysis of 500 most variable genes. Percentage of variance explained by each principal component (PC) is indicated on the corresponding axis.

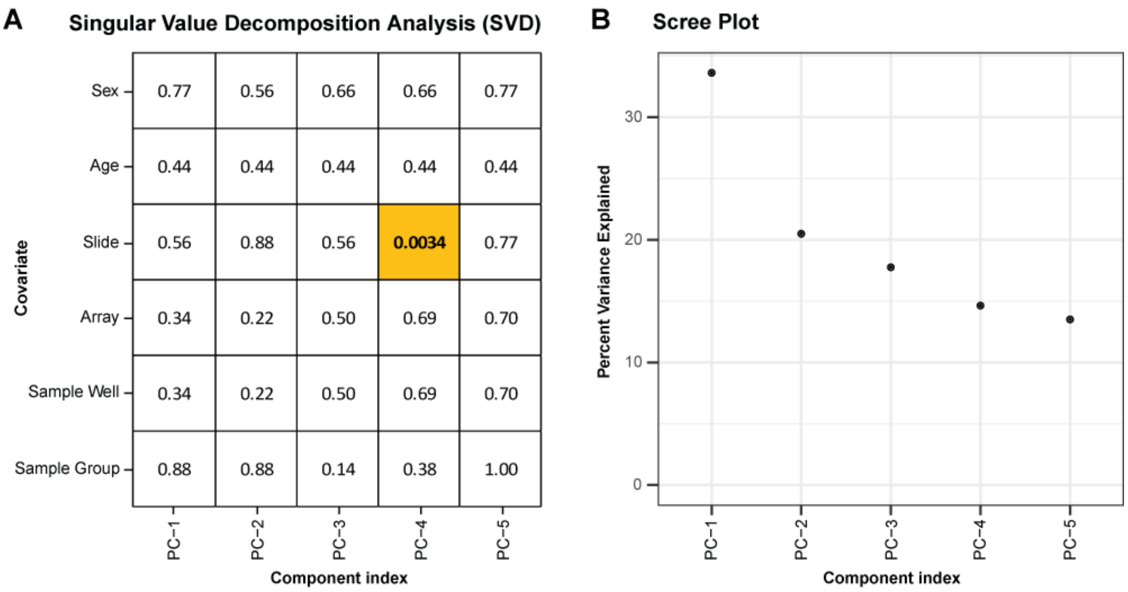

Figure S2. Singular value decomposition analysis (SVD) plots for normalized methylation data from 729969 probes passing filtering. A: matrix of p-values obtained for correlation of each SVD component with each factor of interest. B: scree plot demonstrating percent variance explained by each component.

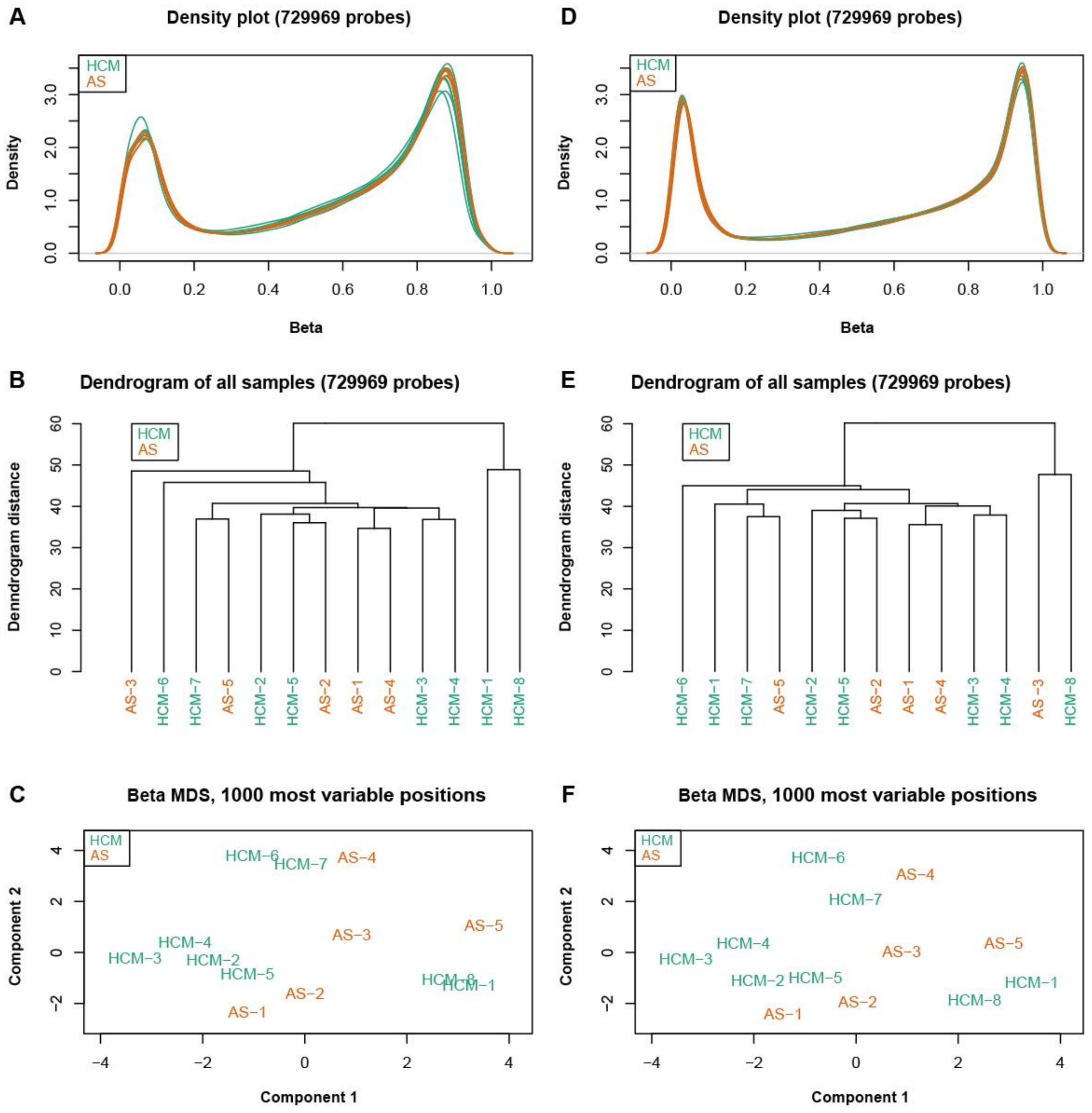

Figure S3. Quality control plots for non-normalized (A-C) and normalized (D-F) methylation data from 729969 probes passing filtering. A,D: the beta distributions for each sample. B,E: the clustering plot for all samples. C,F: multi-dimensional scaling (MDS) of samples according to beta-values of 1000 most variable positions. AS – aortic stenosis, HCM – hypertrophic cardiomyopathy.
